# Analysis of Mucopolysaccharidosis Type VI through Integrative Functional Metabolomics

**DOI:** 10.3390/ijms20020446

**Published:** 2019-01-21

**Authors:** Abdellah Tebani, Lenaig Abily-Donval, Isabelle Schmitz-Afonso, Monique Piraud, Jérôme Ausseil, Farid Zerimech, Carine Pilon, Tony Pereira, Stéphane Marret, Carlos Afonso, Soumeya Bekri

**Affiliations:** 1 Department of Metabolic Biochemistry, Rouen University Hospital, 76000 Rouen, France; abdellah.tebani@yahoo.com (A.T.); Carine.Pilon@chu-rouen.fr (C.P.); 2 Normandie University, UNIROUEN, CHU Rouen, INSERM U1245, 76000 Rouen, France; lenaig.abily-donval@chu-rouen.fr (L.A.-D.); stephane.marret@chu-rouen.fr (S.M.); 3 Normandie Univ, UNIROUEN, INSA Rouen, CNRS, COBRA, 76000 Rouen, France; isabelle.schmitz-afonso@univ-rouen.fr (I.S.-A.); carlos.afonso@univ-rouen.fr (C.A.); 4 Department of Neonatal Pediatrics, Intensive Care and Neuropediatrics, Rouen University Hospital, 76031 Rouen, France; 5 Service de Biochimie et Biologie Moléculaire Grand Est, Unité des Maladies Héréditaires du Métabolisme et Dépistage Néonatal, Centre de Biologie et de Pathologie Est, Hospices Civils de Lyon, 69002 Lyon, France; monique.piraud@chu-lyon.fr; 6 INSERM U1088, Laboratoire de Biochimie Métabolique, Centre de Biologie Humaine, CHU Sud, 80054 Amiens CEDEX, France; jerome.ausseil@chu-amiens.fr; 7 Laboratoire de Biochimie et Biologie Moléculaire, Université de Lille et Pôle de Biologie Pathologie Génétique du CHRU de Lille, 59000 Lille, France; farid.zerimech@chru-lille.fr; 8 Department of Pharmacology, Rouen University Hospital, 76000 Rouen, France; Tony.Pereira@chu-rouen.fr

**Keywords:** metabolomics, inherited metabolic diseases, lysosomal storage diseases, mucopolysaccharidosis type VI, Maroteaux–Lamy syndrome, mass spectrometry

## Abstract

Metabolic phenotyping is poised as a powerful and promising tool for biomarker discovery in inherited metabolic diseases. However, few studies applied this approach to mcopolysaccharidoses (MPS). Thus, this innovative functional approach may unveil comprehensive impairments in MPS biology. This study explores mcopolysaccharidosis VI (MPS VI) or Maroteaux–Lamy syndrome (OMIM #253200) which is an autosomal recessive lysosomal storage disease caused by the deficiency of arylsulfatase B enzyme. Urine samples were collected from 16 MPS VI patients and 66 healthy control individuals. Untargeted metabolomics analysis was applied using ultra-high-performance liquid chromatography combined with ion mobility and high-resolution mass spectrometry. Furthermore, dermatan sulfate, amino acids, carnitine, and acylcarnitine profiles were quantified using liquid chromatography coupled to tandem mass spectrometry. Univariate analysis and multivariate data modeling were used for integrative analysis and discriminant metabolites selection. Pathway analysis was done to unveil impaired metabolism. The study revealed significant differential biochemical patterns using multivariate data modeling. Pathway analysis revealed that several major amino acid pathways were dysregulated in MPS VI. Integrative analysis of targeted and untargeted metabolomics data with in silico results yielded arginine-proline, histidine, and glutathione metabolism being the most affected. This study is one of the first metabolic phenotyping studies of MPS VI. The findings might shed light on molecular understanding of MPS pathophysiology to develop further MPS studies to enhance diagnosis and treatments of this rare condition.

## 1. Introduction

The metabolome can be defined as the complement of all metabolites contained in a given biological system. It embodies the most functional readout of the information embedded in the genotype. Therefore, metabolite changes are usually more suitable to describe the biochemical state of a biological system given their closeness to the phenotype. Given this precious contextual biological information, the metabolome is very appealing to assess pathophysiological states [1,2]. To do so, metabolomics is an “omics” technology that allows the molecular and biochemical characterizations of the metabolome [3,4]. It also describes, holistically, its changes related to both genetic and environmental factors. Inherited metabolic diseases (IMDs) are the most obvious disease group that may directly take advantage of the potential of metabolomics given their underlying pathophysiology [5]. For years, mass spectrometry-based targeted metabolomics have been used in the assessment of IMDs such as aminoacidopathies, organic acidurias, and acylcarnitines for fatty acid oxidation disorders [6,7,8,9,10]. Furthermore, MS is now widely implemented in national inborn errors of metabolism (IEM) newborn screening programs worldwide [11]. Indeed, IMDs are a group of rare diseases mainly related to a genetic defect in enzymes, transporters or cofactors involved in metabolic pathways. Hence, a technology that could infer these impairments in a systematic and holistic fashion is very promising to better understand the metabolic pathways involved in the progression of the disease for better diagnosis and treatment [12,13]. Lysosomal storage disorders (LSDs) represent a group of about 50 inherited disorders related to lysosomal protein deficiencies which cause a progressive accumulation of undegraded metabolites within the lysosome. This metabolite storage leads to various organ failures and premature death [14]. Mucopolysaccharidoses (MPS) is an LSD subgroup due to glycosaminoglycan (GAG) catabolism impairment. Thus, depending on the enzymatic block, one or several GAGs (dermatan sulfate—DS, chondroitin sulfate—CS, heparan sulfate—HS, keratan sulfate—KS, and hyaluronan) may accumulate in lysosomes and the extracellular matrix [15]. The GAG storage causes progressive multiple tissue and organ failures [16]. Eleven known enzyme deficiencies lead to seven distinct forms of MPS [14]. Prenatal symptoms, mainly hydrops fetalis, may be observed in MPS I, MPS IVA, MPS VI, and more frequently in MPS VII [17,18]; however, most MPS patients are asymptomatic after birth. Mucopolysaccharidoses symptoms and severity vary with patients and MPS subtypes. Several MPS treatments are in clinical use or being investigated under clinical trials [19]. Mucopolysaccharidosis VI (MPS VI) or Maroteaux–Lamy syndrome (OMIM #253200) is an autosomal recessive LSD, first described in 1963 [20]. It is caused by the deficiency of the arylsulfatase B (ASB) enzyme also called *N*-acetylgalactosamine 4-sulfatase (E.C.3.1.6.12). This leads to incomplete degradation and accumulation of DS. The arylsulfatase B gene (*ARSB*) is located in chromosome 5 (5q13–5q14) [2].

Mucopolysaccharidoses VI is very rare; its estimated incidence ranges from 0.36 to 1.30 per 100,000 live births [21]. Clinical features, age of onset, and disease progression vary widely in MPS VI patients. A distinction is generally made between slowly and rapidly progressing onsets. However, it is important to note that the phenotype of the disease is rather a clinical continuum. The rapidly progressing phenotype of MPS VI is usually observed before the age of 2 years. The skeleton is generally severely affected with dysostosis multiplex as a typical feature [22]. Musculoskeletal abnormalities can also lead to short stature and low body weight. Other common findings in these patients are coarse facial features [23]. In the absence of treatment, patients with rapidly progressing phenotype generally die from cardiopulmonary disease, infection, or surgical complications with a life expectancy below 20 years. The slowly progressing phenotype of MPS VI is associated with a slower clinical course. The patients show normal or only mildly coarsened facial features, a slightly reduced to normal body height, and less prominent skeletal dysplasia [22,24]. In an advanced stage, patients often develop disability symptoms requiring surgery which lead, ultimately, to a reduction in lifespan [25]. Most MPS VI patients have a poor quality of life due to the high morbidity (impairments of vision, hearing, mobility, and functional capacity along with frequent surgeries), which requires constant and tight management of the disease. Like most rare diseases, for a long time the treatment of MPS VI was limited to palliative care. Recently, targeted therapies have been developed mainly as enzyme replacement therapy Naglazyme™ (Galsulfase, BioMarin) [26,27] and hematopoietic stem cell transplantation (HSCT) [28]. The only available biomarker for MPS VI diagnosis is urinary dermatan sulfate [22]. The aim of this study is to apply both targeted and untargeted metabolic profiling on MPS VI patients, compared to controls, to explore metabolic remodeling in this disease.

## 2. Results

### 2.1. Untargeted Analysis

**Figure 1:**
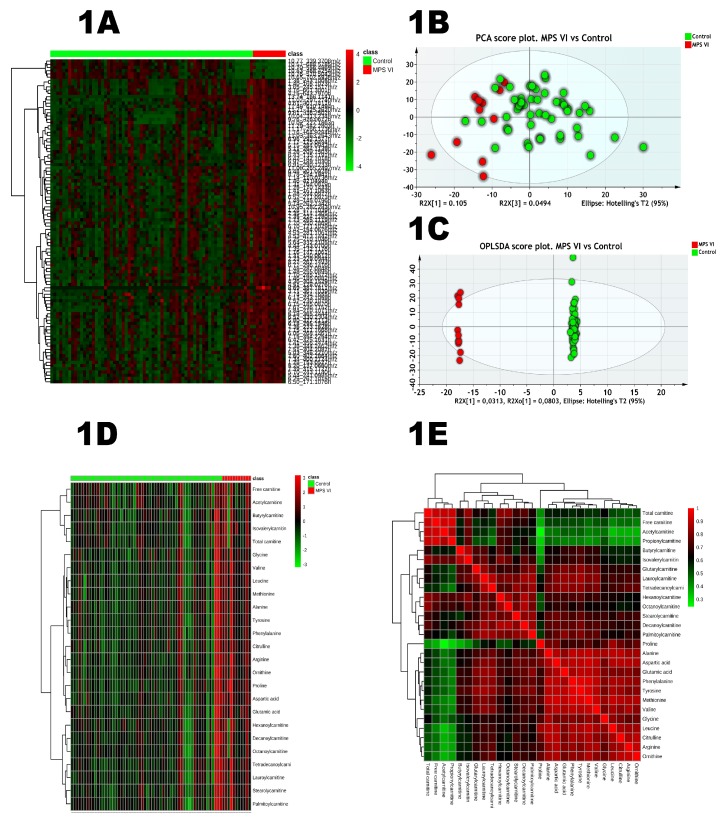
(**A**) Hierarchical cluster analysis and heat map visualization of the top 100 variables (*x*-axis) ranked by *t*-test. The urine sample classes are represented along the *x*-axis. The color code was used to represent log-scaled intensities of features between −4 (green) and +4 (red), showing the features’ relative abundance according to the groups. (**B**) PCA scores’ plot of the normalized dataset. The two groups are represented by different colors. A clear separation is observed between the groups with a clear clustering of the control group. (**C**) OPLSDA scores’ plot (*R2* = 0.99, *Q2*= 0.60) shows a clear separation between the MPS VI and controls. Detailed model characteristics and validation are given in the Supplementary Materials. (**D**) Heat map representing the clustering of amino acids, free and total carnitine along with acylcarnitines across the two groups: Mucopolysaccharidosis (MPS) VI and controls. Columns represent individual samples and rows refer to amino acid. Shades of red or green represent elevation or decrease, respectively, of an amino acid. (**E**) Spearman rank–order correlation matrix assessed targeted metabolites based on their concentration profiles across all samples. Shades of green or red represent low-to-high correlation coefficient between metabolites.

The heatmap in Figure 1A represents the top 100 features ranked by *t*-test (*p* < 0.05 cut-off and FDR 5%) suggesting differential patterns between the study groups and two main clusters are clearly individualized. To explore further, we performed a principal component analysis (PCA) as a dimension reduction tool. PCA is an unsupervised method which is very useful to track clustering trends and identify potential outliers. The PCA score plot also revealed a clustering trend between controls and patients’ samples (Figure 1B). To unveil metabolic features underlying the group separation, supervised methods are more suitable given their predictive characteristics. The OPLS-DA was applied for this classification purpose. Hence, samples were labeled according to their respective groups, control and MPS VI. The final OPLS-DA model had an *R2* = 0.99 and *Q2* = 0.60. The OPLS-DA score plot (Figure 1C) shows that the groups are clearly separated according to their metabolic features. This model is validated by cross-validation using both CV-ANOVA (*p*-value = 2.14 × 10^−6^) and the permutation test (999 permutations). Model validation details are presented in Supplementary Materials Figure S1. Variable importance in projection (VIP) yielded by the built model was used as a variable selection parameter. Based on 1 as a cut-off value, 172 features out of the 854 were selected for the MPS VI vs. the control model. The list of variables was refined by retaining only the most discriminant ones and their putative annotation. Some discriminant features are shown in Table 1 along with their respective annotation accuracy and statistical metrics whereas boxplots and correlation plot with dermatan sulfate are presented in Figure S2. To go further in biological inference, we performed pathway analysis using Mummichog software which is based on mapping significant pathways related to the significantly disturbed metabolite variation. The affected metabolism pathways are shown in Table 2. Interestingly, a series of amino acid and fatty acids pathways are impaired.

### 2.2. Targeted Analysis

**Figure 2:**
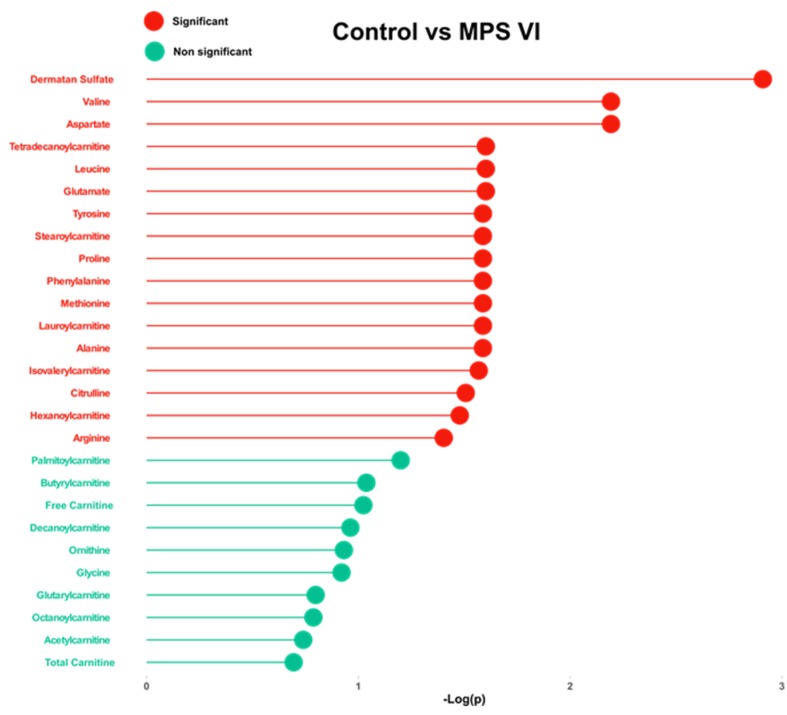
Bar plot showing of dermatan sulfate, total carnitine, free carnitine, and the 13 amino acids and their related −log (*p*) values between MPS VI and controls. Cut-off is set to FDR = 0.05. Corresponding *p*-values are presented in Table 3.

**Figure 3:**
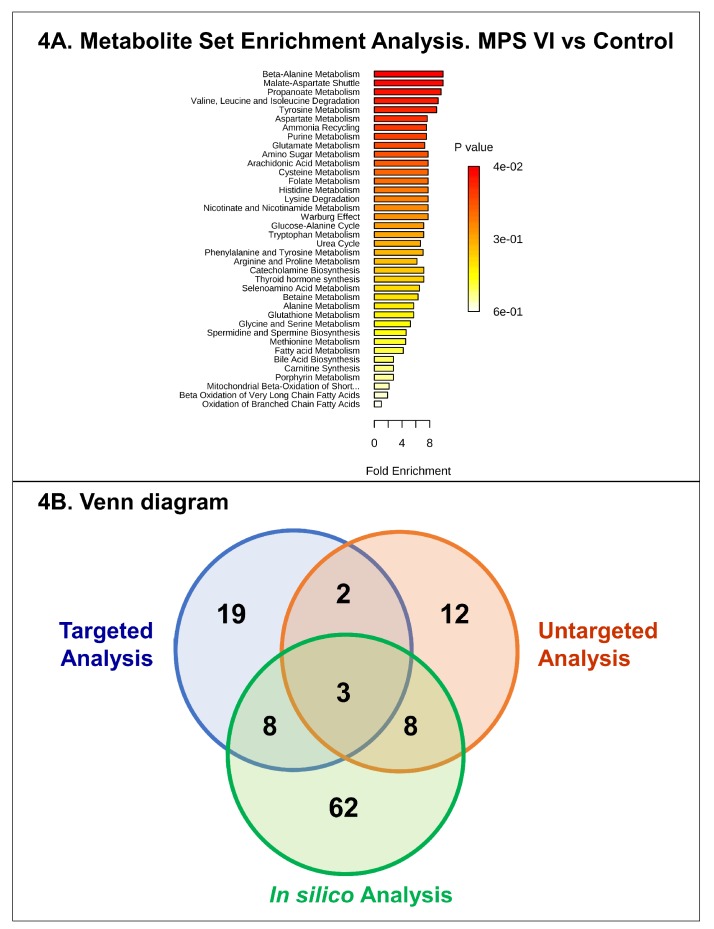
(**A**) Pathway analysis using the assessed free carnitine, acylcarnitines, and the 24 amino-acid concentrations. (**B**) Venn diagram of the significant pathways retrieved from untargeted, targeted approaches, and in silico systems biology approach from Salazar DA et al. [25]. The diagram shows three common metabolisms: glutathione metabolism, histidine metabolism, arginine and proline metabolism. Detailed pathway information is given in the Supplementary Material Table S4.

For targeted analysis, twenty-four amino acids, free carnitine, and twelve acylcarnitines were assessed in all urine samples. Table S1 shows urine concentrations, and boxplots of normalized amino acid concentrations are presented in Figure S3. The statistical analysis results are listed in Table 3 and Figure 2. This differential analysis yielded sixteen metabolites that have shown significant differences highlighting aspartic acid and alanine as the most significant metabolites. A hierarchical clustering analysis has been applied to track sample clustering with similar metabolic profiles. All assessed metabolites, ranked by *t*-test, are presented in the heatmap in Figure 1D. The heatmap shows a clear pattern of sample grouping to respective groups which is also highlighted in the dendrogram structure by its two longest branches; one related mainly to free carnitine and acylcarnitines and the other cluster mainly to amino acids. A correlation analysis of targeted metabolites has been done and the heatmap is depicted in Figure 1E. Both figures show a clear clustering of variables that have high correlation. One includes carnitine and acylcarnitines, and the other amino acids. To assess the diagnostic performance of the different targeted metabolites, univariate ROC curve analyses were used (Table 3). The top metabolites with a high AUC were: aspartic acid (0.85), valine (0.83) and glutamic acid (0.79). A comparison of different classifiers combining the main significant metabolites was performed using both support vector machine (SVM) and PLSDA models with three components each (Figure S4). This combination did not improve the predictive performances of the classifiers. We also performed pathway analysis using these targeted analysis data which yielded as the main impaired metabolisms: aspartate metabolism, glutamate metabolism, valine, leucine and isoleucine degradation, tyrosine metabolism, ammonia recycling and purine metabolism. Salazar et al. [29] generated an in-silico MPS VI model by silencing the *ARSB* gene. The metabolic effects have been analyzed and the altered pathways have been reported [29]. In an attempt to get a deeper insight in our data, we compared the altered pathways identified through our model using experimental metabolomics data to those of Salazar et al.’s [29] in-silico model. As illustrated in detail (Table S2) and Venn diagram (Figure 3B), this comparison showed three main common metabolic pathways: glutathione, histidine and arginine-proline metabolism. The overall results are shown in Figure 3.

## 3. Discussion

This study aimed to investigate MPS VI urine metabolic patterns to unveil the putative biological differences compared to control individuals. The adopted strategy, untargeted metabolomics analysis, enabled us to build a predictive model showing two clusters corresponding to patient and control groups. These differential metabolic patterns are underlined by the major metabolic remodeling in MPS VI group with amino acid-related metabolism being the most affected. The second confirmatory step consisted in quantifying amino acids and acylcarnitines using a targeted approach. Therefore, we performed an integrative analysis between the pathway analysis results of this present study and those of Salazar et al. [29] based on an in-silico strategy by silencing *ARSB* gene. As shown in Figure 3 and Table S5, three main metabolisms are common: arginine-proline, histidine, and glutathione metabolism.

It is now admitted that the clinical manifestations of lysosomal storage diseases are only partly explained by the adverse effects of the lysosomal non-degraded material and that other common pathogenic effects underlie LSDs [30]. In the case of MPSs, the GAG storage affects key cellular processes, and major signaling pathways such as Sonic hedgehog (Shh) and Wnt/β-catenin signaling. These pathways are involved in both morphogenetic processes and tissue homeostasis [31,32,33]. This GAG-related effect is worsened by pathophysiological disruption common to all LSDs such as impairment of autophagy and mitophagy processes responsible for cell damages and death. The subsequent increase of altered mitochondria and reactive oxygen species may represent the main pathogenic mechanism of LSD diseases [34]. In this context, it is instructive to note that the main altered pathways characterized in this study, arginine-proline, histidine and glutathione metabolisms, are involved in regulating autophagy and protecting from oxidative stress. The mechanistic target of rapamycin complex 1 (mTORC1) is a key nutrient/energy sensor; the active form of mTORC1 promotes anabolic biosynthetic pathways and inhibits catabolic processes mainly autophagy. The activation/inactivation of mTORC1 is dependent on its intracellular localization. It has been well documented that amino acids are involved in mTORC1 regulation/localization and this amino acid-sensing localization occurs both in the lysosome and the cytoplasm. Arginine is the main amino acid contributor to this regulation. The alteration of arginine pathway in LSDs may represent a major cause of autophagy deregulation. Of note, metabolomics analysis of urine MPS I and MPSIII patients versus controls have also demonstrated a major alteration of arginine pathway [35,36]. Histidine and glutathione pathways are linked to oxidative stress process and have both protective effects toward reactive oxygen species (ROS). Histidine is the precursor of carnosine (β-alanyl-l-histidine). The latter is likely to exert a protective effect in oxidative diseases. Histidine and carnosine are active through the imidazole cycle of histidine molecule that helps in scavenging ROS [37,38]. Autophagy dysfunction exacerbates cellular stress generation and in turn, the increased ROS production triggers the alteration of both autophagy and apoptosis processes. Accordingly, recent studies unveiled the emerging common cellular pathways involved upon oxidative stress that link autophagy and apoptosis [39]. Mitochondria dysfunction and the subsequent programed cell death disruptions may represent the most significant cellular deregulation underlying LSD pathogenesis. Indeed, oxidative damage contribution in the LSD pathophysiology in general and in MPSs in particular has recently been documented [34,40]. These data suggest metabolic remodeling and provide a functional snapshot profile of metabolite abundances. More mechanistic studies are needed for deeper exploration of these disturbances. Furthermore, this study presents some limits regarding: (i) the small number of patients mainly related to the rarity of the disease, and (ii) age distribution heterogeneity related to the stringent conditions for control of pediatric cohorts; this has been addressed using a covariate correction method (limma R-package). Given the broader scope of mass spectrometry-based untargeted metabolomics regarding metabolome coverage, the presented results are still exploratory, yet encouraging, and require further validation studies through more mechanistic experiments to go deeper in understanding the role of the unveiled metabolisms and their interactions with dermatan sulfate metabolism.

## 4. Material and Methods

### 4.1. Urine Samples

Since the MPS VI diagnosis is mainly based on urine analysis, urine samples have been widely included in expert centers collections. Random morning urine samples were collected from MPS VI patients with confirmed diagnosis by demonstrating marked enzyme deficiency in leucocytes and/or by molecular analysis. Urine samples were collected in four expert centers for inherited metabolic diseases in France. Sixteen MPS VI patients were evaluated: 10 males (age range from 3.1 to 14.7 years, mean age: 6.9 years) and six females (age range from 1.6 to 11.7 years, mean age: 3.9 years). Control urine samples were from 66 healthy subjects, 27 males and 39 females (age range from 5.5 to 70 years, mean age: 40.8 years). The samples were retrieved from biological collections authorized by the French Ministry of Research. The samples were obtained in accordance with Good Clinical Practices. This project was approved by the Research Ethics Board of Rouen University Hospital (CERNI 27-04-2016 E2016-21).

### 4.2. Reagents and Chemicals

Acetonitrile was purchased from VWR Chemicals (Fontenay-sous-Bois, France), ultrapure water (18 MX) from Millipore (Molsheim, France) and formic acid from Fluka (Saint Quentin Fallavier, France). The chemicals used were of analytical grade. Leucine Enkephalin (Saint-Quentin Fallavier, Sigma–Aldrich, France) at a concentration of 2 ng/mL (in acetonitrile/water, 50/50) was used as reference for mass measurements. Poly-DL-alanine was prepared in 50:50 (*v*/*v*) water/acetonitrile at 10 mg/L and used for ion mobility cell calibration. MassChrom^®^ Amino Acid and Acylcarnitine kit (Ref. 55000) was purchased from Chromsystems (Gräfelfing Germany). HPLC gradient grade methanol was purchased from VWR Chemicals. Dermatan sulfate calibration standard was from Sigma–Aldrich (Saint-Quentin Fallavier, France).

### 4.3. Untargeted Metabolic Phenotyping

**Figure 4:**
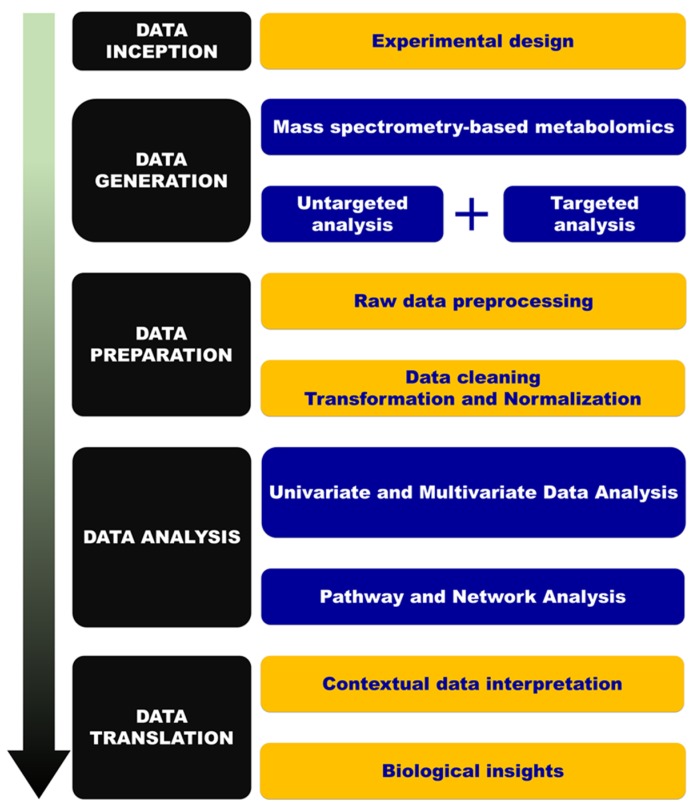
Illustration of the metabolomics workflow spanning from experimental design to pathway analysis and biological interpretation. HMDB: Human Metabolome Database. KEGG: Kyoto Encyclopedia of Genes and Genomes. MetCCS: Metabolite CCS database. MSEA: Metabolite Set Enrichment Analysis. RSD: relative standard deviation.

The protocol used in this study was previously described [35] and Figure 4 presents an overview of the implemented metabolomics workflow.

#### 4.3.1. Sample Handling

The sample handling component was a Waters 2777C sample manager (Waters Corp., Milford, MA, USA) equipped with a 25 μL Hamilton syringe, a 2 μL loop used for full loop injections of prepared sample, and a 2-drawer sample chamber thermo-stated at 4 °C with a constant flow of dry nitrogen gas to prevent the buildup of condensation.

#### 4.3.2. Chromatographic Conditions

The chromatography was performed on a Waters NanoAcquity UPLC module (Saint Quentin en Yvelines, France) upgraded to work with a 1-mm column and composed with a binary solvent manager and column heater/cooler module. Separation was carried out at 45 °C using a 1.0 × 100 mm, Acquity UPLC HSS T3 column (Waters), with a particle size of 1.8 µm, equipped with a 0.2-µm prefilter. Urine was eluted from the LC column using the following linear gradient (curve number 6): 0–1 min: 99% A; 1–3 min, 99–85% A; 3–6 min, 85–50% A; 6–9 min, 50–0% A; 9–12 min, 100% B, 12–16 min, 99% A for re-equilibration. Solvent A was water and solvent B was acetonitrile, both solvents contained 0.1% formic acid. Sample analysis order has been randomized to avoid potential for confounding critical variables with analytical run order effects.

#### 4.3.3. Ion Mobility and Mass Spectrometry

The U-HPLC system was coupled to a hybrid quadrupole orthogonal time-of-flight (TOF) mass spectrometer (SYNAPT G2 HDMS, Waters MS Technologies, Manchester, UK). The mass spectrometer was operated in positive electrospray ionization mode. A mass range of *m*/*z* 50−1200 was used in both modes. The sample cone voltage, extraction cone voltage, source temperature, desolvation temperature, desolvation gas flow, and cone gas flow were optimized and were as follows respectively: 25 V, 5 V, 120 °C, 500 °C, 400 L/h, 50 L/h. Leucine enkephalin was used as the lock mass [M + H]^+^ at *m*/*z* 556.2771. Sodium formate solution was used for external instrument calibration. The Synapt G2 HDMS was equipped with a traveling wave “Triwave™” geometry in which the ion mobility cell (IMS T-wave). The helium cell gas flow, wave height, Trap Bias, and IMS wave delay were set at 180 mL/min, 40 V, 45 V, and 450 µs, respectively. The TOF analyzer was operated in the V resolution mode with an average mass resolution of m/Δm 20,000 (full-width at half-maximum definition). Data acquisition of an ion mobility experiment consisted of 200 bins. The CCS values, obtained in nitrogen, were experimentally determined using singly charged Poly-DL-alanine oligomers as the TWIM calibrant species for ESI+. The CCS values were derived according to previously reported procedures [41]. The ion mobility resolution was ~40 Ω/ΔΩ (fwhm). The N2 CCS values reported were determined at the apex of the ion-mobility peak.

#### 4.3.4. Raw Data Processing

All LC-IM–MS raw data files data processing, peak detection and peak matching across samples using retention time (*t*_R_) correction and chromatographic alignment along with drift time and CCS calculation were performed using Progenesis QI (Waters MS Technologies, Manchester, UK) to yield a data matrix containing retention times, accurate masses, CCS, and peak intensities. The preprocessing step resulted in an X-matrix where tR, CCS and *m*/*z* values were concatenated into “*t*_R_ _*m*/*z*_CCS” features (in columns) present in each sample (in rows) with corresponding peak areas.

#### 4.3.5. Quality Control

Aliquoted 10 μL of each urine sample were mixed together to generate pooled quality control samples (QCs). The QCs and solvent blank samples (mobile phase) were injected sequentially in-between the urine samples. In addition, a dilution series of QC samples (6%, 12.5%, 25%, 50% and 100% original concentration) were used to assess the quality of the extracted features. In this study, we used a filter strategy in which the features intensity must be correlated to the matrix concentrations in a series of diluted QC samples in order to be included in further analysis. Feature groups with correlation coefficient of less than 0.70 were removed from the dataset. Furthermore, datasets were refined by removal of feature groups that did not meet the threshold of peak area measurement precision prior to data analysis. This approach uses RSD values derived from repeated measurements of a pooled QC sample. The threshold was set to RSD < 25% to enhance the biological interpretation of metabolomics data.

### 4.4. Targeted Analysis

#### 4.4.1. Amino Acids and Acylcarnitines Profiling

Amino acids, free carnitine, and acylcarnitines were semi-quantified using a flow injection tandem mass spectrometry method based on a Masschrom^®^ amino acids and acylcarnitines kit (chromsystems, gräfelfing germany). The analyses were performed following the instructions of the kit manufacturer using liquid chromatography instrument prominence Shimadzu UFLC System (Shimadzu, Prominence, Kyoto, Japan) coupled to the 4000 Qtrap mass spectrometer (Sciex, Framingham, MA, USA) with an electrospray ion source. A system suitability test was conducted before each batch of the samples (analysis of a standard mixture) to warm up the LS-MS/MS system and check the inter-day performance of the system. Data acquisition and processing were performed using the Analyst 1.5 software (Sciex, Framingham, MA, USA). The list of measured MRM transitions is presented in Tables S3 and S4.

#### 4.4.2. Dermatan Sulfate Assessment

Dermatan sulfate was assessed as previously described [42]. Briefly, 25 μL of homogenized urine sample was evaporated under a nitrogen stream. Then, 500 μL of a commercial methanolic HCl 3 N solution was added and vortexed. The samples were incubated at 65 °C for 1 h, then immediately evaporated under a nitrogen stream and then suspended in 200 μL of the resuspension solution containing dermatan sulfate (9 μg/mL) 90:10 acetonitrile:water solution. Samples were transferred to vials and centrifuged prior to the injection of 2 μL in the HPLC-MS/MS system.

### 4.5. Data Analysis and Modeling

Support vector regression normalization method was applied using the MetNormalizer R package [43] before any data analysis of untargeted metabolomics data to remove the unwanted intra- and inter-batch measurement analytical variations. Then the normalized data matrix was log-transformed and pareto-scaled. All data analyses and modeling were done using SIMCA 14.0 (MKS DAS, Umeå, Sweden). First, hierarchical cluster analysis was applied to the dataset to get an overview of the clustering trends of samples with similar profiles of variable intensity. Furthermore, multivariate data analysis and modeling was performed using principal component analysis (PCA) as an unsupervised method where the variations in correlated variables were summarized into a smaller number of latent variables, so-called principal components. The latent variables were used to describe the observations. The relationship between observations that are characterized by many variables can be visualized in low dimensional plots. This is what makes PCA a dimension reduction method to better visualize high numbers of variables. Principal component analysis was first applied to get an overview of the data and identify potential severe outliers which were defined as observations whose scores mapped outside the Hotelling’s T2 ellipse (confidence interval = 0.95) in a cross-validated seven-component model [44]. Orthogonal partial least-squares-discriminant analysis (OPLS-DA) was used as a supervised method. This method aims to maximize the covariance between the independent variables (metabolites) and the corresponding dependent variable Y (groups, i.e., Control vs Disease) by finding a linear subspace of the explanatory variables. This space allows the prediction of the Y variable (groups) based on a reduced number of factors (PLS components). This method provides several statistical metrics such as variable importance on projection (VIP) which is a parameter used for calculating the cumulative measure of the influence of individual X-variables on the model, and therefore the discriminative power of each variable (metabolite). To select the most relevant features, the training group was repeatedly been split into a training set and a test set. A permutation test (999 iterations) was performed to prevent the OPLS-DA over fitting of the model by comparing diagnostic statistic metrics of the generated model with those of randomly generated models. R2X is the cumulative modeled variation in X (X = features), R2Y is the cumulative modeled variation in Y (Y = sample groups), and Q2Y is the cumulative predicted variation in Y, based on the cross-validation. The range of these parameters was between 0 and 1, where 1 indicates a perfect fit. Furthermore, cross-validated analysis of variance (CV-ANOVA) was systematically performed based on the cross-validated model [45]. The X matrix was 82 × 854 variables. For all differential analysis, confounding factor effects (gender and age) correction was made using the limma package [46]. Given the high number of tested metabolites, the probability of finding a random association between a given metabolite and the group increases. This accumulation of false positives is called the multiple testing problem when a statistical test is applied across multiple features. Therefore, retrieved *p*-values from multiple tests performed in parallel across the metabolites should be corrected. Different methods are proposed to handle this issue. We used the false discovery rate method with 0.05% as the cut-off. Sensitivity and specificity are two basic measures of diagnostic accuracy. Sensitivity, or true-positive probability (TPP), is the probability of test results being positive for actually abnormal cases. Specificity, or true-negative probability (TNP), is the probability of test results being negative for actually control cases. The receiver operating characteristic (ROC) curve is a plot of TPP versus its false-positive fraction (FPF), or 1-specificity. Thus, the ROC curve is a valuable tool to assess and compare the diagnostic accuracy between biomarkers [47]. The area under the entire curve (AUC) is widely used as a summary metrics of the ROC curve. The AUC might be seen as an average value of sensitivity for all possible values of specificity. Thus, the greater the AUC, the better is the test. A perfect biomarker would have 100% sensitivity and 100% specificity. The point on the ROC curve which corresponds to this perfect case would be at the upper left-hand corner (0, 1) which lead to an AUC = 1 while a useless biomarker would have AUC = 0.5. Most of biomarkers fall between these two values.

### 4.6. Feature Selection, Annotation, and Network Analysis

To select the most discriminant variables for the separation of groups we use the covariances and correlations between the X matrix and OPLS scores. The covariance values give the magnitude of the contribution of a variable while the correlation values reflect the effect and reliability of the variable for the model component scores. Variables with both very high correlation and covariance are important for the model’s explanation. Furthermore, selection of discriminant variables was achieved using the VIP score procedures for each validated OPLS-DA model [48]. Putative annotation of detected features was performed using accurate mass comparison using freely available metabolite databases HMDB, LipidBlast, and Metlin. Furthermore, CCS values were also compared to the MetCCS database [49]. In order to provide a broader understanding of metabolic changes in MPS VI, we also explored the biochemical pathways using a network analysis approach using Mummichog (v.1.0.5) which allows pathway enrichment analyses. The idea behind this metabolic network prediction strategy assumes that metabolite concentration alterations are more likely to occur within a metabolic connected network rather than in a random fashion. This Mummichog Python package highlights pathways that are significantly impacted in the studied groups. Significantly impacted biochemical pathways are those exhibiting an adjusted *p*-value < 0.05. For this comparison, we focused on features that significantly changed (511 features with *q*-values = 0.05 and FDR = 5%). Mummichog annotates metabolites based on accurate mass *m*/*z* (5 ppm mass error was used) and tests significant pathway enrichment within a reference metabolic network using a Fisher’s exact test [50]. The matched candidates were then mapped to reference human metabolic networks from the KEGG, MetaCyc, Recon, and Edinburgh Human Metabolic Network. The null distribution in pathway analysis was obtained from 1000 set of randomly permutated m/z lists draw from all features detected in the whole metabolomic dataset and modeled by Gamma distribution. To protect against incorrect pathway selection, redundant pathways or those enriched by fewer than two metabolites were excluded. MetaboAnalyst [51] has been used for metabolite set enrichment analysis using the amino acid concentration matrix.

## 5. Conclusions

Profound metabolic remodeling beyond the targeted deficient pathway is unveiled using an integrative untargeted and targeted metabolomics approach to investigate urine samples originating from MPS VI patients. This study revealed the alteration of different metabolisms seemingly without any apparent link with the metabolism of dermatan sulfate, although there are some studies showing a link between glycosaminoglycans and lipid metabolism, including lipid deposits in the case of GAG accumulation [52]. This shows the importance of such global omics studies to reveal possible links between different nodes in a metabolic network that could not be revealed with reductionist strategies. This crucial information is needed for a holistic understanding of functional mechanisms underlying this rare condition. More targeted validation studies are required to explore the impaired metabolism at a mechanistic level. This may end up to a better management of these patients by tuning these altered pathways through autophagy modulation and antioxidant adjuvants which might potentiate MPS specific therapies.

## Figures and Tables

**Table 1:** Some discriminant putatively annotated features and related statistical metrics.

HMDB	Putative Annotation	Formula	*M*	*m*/*z*	Adduct	Δ*m*/*z* (ppm)	*t*_R_ (min)	*t*_D_ (ms)	CCS (A^2^)	%RSD	VIP	FDR	AUC
HMDB0003464	4-guanidinobutanoic acid	C_5_H_11_N_3_O_2_	145.0851	146.0932	M + H	5	1.48	1.89	124.4	4.60	0.97	5.18 × 10^−4^	0.88
HMDB0001276	*N*-acetylspermidine	C_9_H_21_N_3_O	187.1685	188.1774	M + H	9	1.25	2.38	139.3	8.58	0.53	1.74 × 10^−2^	0.83
HMDB00062	Carnitine	C_8_H_18_N_4_O_2_	202.1430	203.1518	M + can + H	0.48	1.41	2.43	140.4	9.97	0.70	3.52 × 10^−3^	0.85
HMDB0015444	Phenylalaninylalanine	C_12_H_16_N_2_O_3_	236.1161	237.1225	M + H	4	7.67	2.7	147.8	10.51	1.55	1.95 × 10^−4^	0.94
HMDB0002012	Ubiquinone-1	C_14_H_18_O_4_	250.1205	251.1291	M + H	5	7.17	2.86	152.4	5.69	0.16	2.74 × 10^−2^	0.80
HMDB0000145	Estrone	C_18_H_22_O_2_	270.1620	271.1675	M + H	6	6.50	3.19	161.5	4.26	0.27	2.83 × 10^−2^	0.79

M: monoisotopic mass, ppm: parts per million; *t*_R_: retention time; *t*_D_: drift time; CCS: cross collision section; VIP: variable importance in projection; RSD: relative standard deviation; FDR; false discovery rate; AUC: area under curve.

**Table 2:** Significantly dysregulated pathways.

Pathway	Overlap Size	*p*-Value (FDR = 5%)
Vitamin B9 (folate) metabolism	5	2.87 × 10^−4^
Glycine, serine, alanine and threonine metabolism	7	3.36 × 10^−4^
Alanine and Aspartate metabolism	4	4.68 × 10^−4^
Histidine metabolism	4	1.29 × 10^−3^
Vitamin E metabolism	5	2.21 × 10^−3^
Carnitine shuttle	5	2.21 × 10^−3^
Glycosphingolipid metabolism	3	3.61 × 10^−3^
Vitamin B3 (nicotinate and nicotinamide) metabolism	3	3.61 × 10^−3^
Selenoamino acid metabolism	2	4.15 × 10^−03^
Glutathione Metabolism	2	4.15 × 10^−3^
CoA Catabolism	2	4.15 × 10^−3^
Electron transport chain	2	4.15 × 10^−3^
Vitamin B5–CoA biosynthesis from pantothenate	2	4.15 × 10^−3^
Methionine and cysteine metabolism	6	4.66 × 10^−3^
Aspartate and asparagine metabolism	7	8.25 × 10^−3^
Purine metabolism	5	1.01 × 10^−2^
Arginine and proline metabolism	4	1.20 × 10^−2^
Lysine metabolism	4	1.70 × 10^−2^
Linoleate metabolism	4	1.70 × 10^−2^
Aminosugar metabolism	3	2.29 × 10^−2^
Porphyrin metabolism	3	2.29 × 10×^2^
Pyruvate metabolism	2	2.63 × 10^−2^

FDR: False discovery rate.

**Table 3:** The normalized concentrations of dermatan sulfate, free amino acids, carnitine (total and free), and acylcarnitines in urine samples of the MPS VI and control groups. (μM/mM creatinine).

	Control vs. MPS VI			
	AUC	*p*-Value (FDR)	Fold Change	Effect in MPS VI
Dermatan sulfate	0.90	1.23 × 10^−3^	10.0	Increased
Aspartic acid	0.85	6.41 × 10^−3^	1.61	Increased
Valine	0.83	6.41 × 10^−3^	1.74	Increased
Glutamic acid	0.79	2.50 × 10^−2^	1.42	Increased
Leucine	0.79	2.50 × 10^−2^	1.44	Increased
Tetradecanoylcarnitine	0.78	2.50 × 10^−2^	1.36	Increased
Alanine	0.75	2.58 × 10^−2^	1.32	Increased
Lauroylcarnitine	0.75	2.58 × 10^−2^	1.18	Increased
Methionine	0.77	2.58 × 10^−2^	1.28	Increased
Phenylalanine	0.77	2.58 × 10^−2^	1.28	Increased
Proline	0.79	2.58 × 10^−2^	1.48	Increased
Stearoylcarnitine	0.76	2.58 × 10^−2^	1.07	Increased
Tyrosine	0.76	2.58 × 10^−2^	1.30	Increased
Isovalerylcarnitine	0.71	2.70 × 10^−2^	1.42	Increased
Citrulline	0.79	3.11 × 10^−2^	1.26	Increased
Hexanoylcarnitine	0.71	3.32 × 10^−2^	1.20	Increased
Arginine	0.80	3.95 × 10^−2^	1.52	Increased
Palmitoylcarnitine	0.68	6.31 × 10^−2^	0.00	/
Butyrylcarnitine	0.68	9.16 × 10^−2^	1.15	Increased
Free carnitine	0.67	9.46 × 10^−2^	1.27	Increased
Decanoylcarnitine	0.67	1.09 × 10^−1^	0.90	Decreased
Ornithine	0.75	1.17 × 10^−1^	1.05	Increased
Glycine	0.70	1.20 × 10^−1^	0.95	Decreased
Glutarylcarnitine	0.66	1.59 × 10^−1^	0.95	Decreased
Octanoylcarnitine	0.65	1.63 × 10^−1^	0.79	Decreased
Acetylcarnitine	0.62	1.82 × 10^−1^	1.04	Increased
Total carnitine	0.62	2.02 × 10^−1^	0.89	Decreased

## Supplementary Materials

Supplementary materials can be found at http://www.mdpi.com/1422-0067/20/2/446/s1.

## Abbreviations

IEM	Inborn errors of metabolism
LSD	Lysosomal storage diseases
MPS	Mucopolysaccharidoses
GAGs	Glycosaminoglycans
MPS VI	Mucopolysaccharidosis type VI
ERT	Enzyme replacement therapy
HS	Heparan sulfate
CCS	Collision cross section
DS	Dermatan sulfate
KS	Keratan sulfate
ROC	Receiver operating characteristic
FDR	False discovery rate
PCA	Principal component analysis
OPLS-DA	Orthogonal partial least-squares-discriminant analysis
VIP	Variable influence in projection
AUC	Area under curve
