# Supplementary material for: Analysis of Mucopolysaccharidosis Type VI through Integrative Functional Metabolomics
Source: Int J Mol Sci. 2019 Jan 21;20(2):446. doi: 10.3390/ijms20020446 (PMC6359186; doi:10.3390/ijms20020446)
Supplement: Supplementary file 1 [file ijms-20-00446-s001.pdf]

# Supplementary material for:

# Analysis of mucopolysaccharidosis type VI through integrative functional metabolomics

**Abdellah Tebani[1,2,3], Lenaig Abily-Donval[2,4], Isabelle Schmitz-Afonso[3], Monique Piraud[5], Jérôme Ausseil[6], Farid Zerimech[7], Carine Pilon[1], Tony Pereira[8], Stéphane Marret[2,4], Carlos Afonso[3], Soumeya Bekri[1, 2,*]**

[1] Department of Metabolic Biochemistry, Rouen University Hospital, Rouen, 76000, France
[2] Normandie Univ, UNIROUEN, CHU Rouen, INSERM U1245, 76000 Rouen, France
[3] Normandie Univ, UNIROUEN, INSA Rouen, CNRS, COBRA, 76000 Rouen, France
[4] Department of Neonatal Pediatrics, Intensive Care and Neuropediatrics, Rouen University Hospital, Rouen, 76031, France
[5] Service de Biochimie et Biologie Moléculaire Grand Est, Unité des Maladies Héréditaires du Métabolisme et Dépistage Néonatal, Centre de Biologie et de Pathologie Est, Hospices Civils de Lyon, France.
[6] INSERM U1088, Laboratoire de Biochimie Métabolique, Centre de Biologie Humaine, CHU Sud, 80054, Amiens Cedex, France.
[7] Laboratoire de Biochimie et Biologie Moléculaire, Université de Lille et Pôle de Biologie Pathologie Génétique du CHRU de Lille, 59000, Lille, France.
[8] Department of Pharmacology, Rouen University Hospital, 76000, Rouen, France.

**Corresponding author:**
Prof. Soumeya BEKRI
Department of Metabolic Biochemistry,
Rouen University Hospital 76031 ROUEN Cedex France
soumeya.bekri@chu-rouen.fr
Tel 00 33 2 32 88 81 24
Fax 00 33 2 32 88 83 41

# Contents

## Tables

**Table S1.** MRM transitions for free carnitine and acylcarnitines and the corresponding internal standard.
**Table S2.** MRM transitions for amino acids and the corresponding internal standard.
**Table S3.** The normalized concentrations of free amino acids, carnitine (total and free) and acylcarnitines in urine samples of the MPS VI and control groups.
**Table S4.** Data for Venn diagram of the significant pathways retrieved from untargeted, targeted approaches and *in silico* systems biology.

## Figures

**Figure S1.** OPLSDA model validation including the two groups: MPS VI and Controls.
**Figure S2.** Boxplots of selected discriminant features in the assessed groups: MPS VI and Control samples.
**Figure S3.** Boxplots of amino acid, carnitine (free and total) and acylcarnitine concentrations.
**Figure S4** Area under the receiver operating characteristic (ROC) curves, comparing diagnostic performance of the most significant quantified metabolites to differentiate MPS VI from Control samples.

**Table S1. The normalized concentrations of amino acids (μM/mM creatinine), carnitine (total and free) and acylcarnitines (μM/mM creatinine) in urine samples of from MPS VI and control groups.**

| | Control | | MPS VI | |
|---|---|---|---|---|
| | **Mean** | **Range** | **Mean** | **Range** |
| **Alanine** | 4.645 | 0.682 - 101 | 10.649 | 3.381 - 68.8 |
| **Arginine** | 0.811 | 0.095 - 287.736 | 2.138 | 0.631 - 18.6 |
| **Aspartic acid** | 0.998 | 0.198 - 13.6 | 2.802 | 1.333 - 14.2 |
| **Citrulline** | 0.189 | 0.035 - 3.278 | 0.415 | 0.187 - 1.3 |
| **Glutamic acid** | 3.727 | 0.322 - 68.7 | 9.169 | 3.183 - 58.2 |
| **Glycine** | 26.339 | 3.884 - 552 | 46.758 | 15.357 - 446 |
| **Leucine** | 1.152 | 0.154 - 18.4 | 2.884 | 1.286 - 14.2 |
| **Methionine** | 0.333 | 0.064 - 8.09 | 0.745 | 0.289 - 4.5 |
| **Ornithine** | 1.378 | 0.258 - 172.17 | 2.621 | 0.945 - 15.5 |
| **Phenylalanine** | 1.051 | 0.094 - 18.2 | 2.355 | 0.844 - 16.8 |
| **Proline** | 0.444 | 0.119 - 12.335 | 1.138 | 0.475 - 9.1 |
| **Tyrosine** | 4.035 | 0.805 - 76.2 | 9.124 | 2.857 - 62.2 |
| **Total carnitine** | 1.214 | 0.031 - 49.693 | 2.066 | 0.173 - 11.384 |
| **Free carnitine** | 9.177 | 0.27 - 358.277 | 20.402 | 2.542 - 120.409 |
| **Acetylcarnitine** | 0.507 | 0.01 - 15.846 | 0.961 | 0.068 - 6.21 |
| **Propionylcarnitine** | 0.034 | 0 - 32.28 | 0.043 | 0.002 - 0.46 |
| **Butyrylcarnitine** | 0.150 | 0.001 - 3.22 | 0.308 | 0.025 - 1.357 |
| **Isovalerylcarnitine** | 0.059 | 0.001 - 0.8 | 0.146 | 0.01 - 0.814 |
| **Glutarylcarnitine** | 0.173 | 0.01 - 7.13 | 0.300 | 0.039 - 2.19 |
| **Hexanoylcarnitine** | 0.012 | 0.001 - 0.146 | 0.025 | 0.003 - 0.115 |
| **Octanoylcarnitine** | 0.020 | 0.002 - 0.328 | 0.032 | 0.005 - 0.192 |
| **Decanoylcarnitine** | 0.005 | 0 - 0.085 | 0.010 | 0.002 - 0.055 |
| **Lauroylcarnitine** | 0.009 | 0.001 - 0.126 | 0.020 | 0.006 - 0.13 |
| **Tetradecanoylcarnitine** | 0.007 | 0.001 - 0.094 | 0.018 | 0.006 - 0.123 |
| **Palmitoylcarnitine** | 0.006 | 0.001 - 0.099 | 0.012 | 0.004 - 0.108 |
| **Stearolycarnitine** | 0.002 | 0 - 0.018 | 0.003 | 0.001 - 0.021 |

**Table S2. Data for Venn diagram of the significant pathways retrieved from metabolomics approaches and *in silico* systems biology approach from Salazar DA et al [1].**

| Data | N° | Pathways |
|---|---|---|
| MPS VI Targeted *vs* MPS VI *in silico vs* MPS VI untargeted | 3 | Glutathione metabolism<br>Histidine metabolism<br>Arginine and proline metabolism |
| MPS VI Targeted *vs* MPS VI untargeted | 2 | Selenoamino acid metabolism<br>Purine metabolism |
| MPS VI Targeted *vs* MPS VI *in silico* | 8 | Urea cycle<br>Glutamate metabolism<br>Cysteine metabolism<br>Tyrosine metabolism<br>Tryptophan metabolism<br>Folate metabolism<br>Beta-alanine metabolism<br>Propanoate metabolism |
| MPS VI *in silico vs* MPS VI untargeted | 8 | Pyruvate metabolism<br>Lysine metabolism<br>Glycine, serine, alanine and threonine metabolism<br>Glycosphingolipid metabolism<br>Alanine and aspartate metabolism<br>CoA catabolism<br>Glycerophospholipid metabolism<br>Methionine and cysteine metabolism |
| MPS VI targeted | 19 | Thyroid hormone synthesis<br>Aspartate metabolism<br>Catecholamine biosynthesis<br>Nicotinate and nicotinamide metabolism<br>Methionine metabolism<br>Fatty acid metabolism<br>Amino sugar metabolism<br>Glycine and serine metabolism<br>Ammonia recycling<br>Lysine degradation<br>Glucose-alanine cycle<br>Betaine metabolism<br>Warburg effect<br>Alanine metabolism<br>Spermidine and spermine biosynthesis<br>Malate-aspartate shuttle<br>Phenylalanine and tyrosine metabolism<br>Valine, leucine and isoleucine degradation<br>Arachidonic acid metabolism |
| MPS VI untargeted | 12 | Linoleate metabolism<br>Urea cycle/Amino group metabolism<br>Porphyrin metabolism<br>Vitamin B5 - coa biosynthesis from pantothenate<br>Pyrimidine metabolism<br>Vitamin B9 (folate) metabolism<br>Amino-sugar metabolism<br>Vitamin B3 (nicotinate and nicotinamide) metabolism<br>Aspartate and asparagine metabolism<br>Vitamin E metabolism<br>Electron transport chain<br>Carnitine shuttle |
| MPS VI *in silico* | 62 | Transport, peroxisomal; Keratan sulfate degradation; Oxidative phosphorylation; Vitamin D metabolism; Purine catabolism; Starch and sucrose metabolism; Pyrimidine catabolism; Amino sugar metabolism; Eicosanoid metabolism; Transport, endoplasmic reticular; Transport, Golgi apparatus; Transport, extracellular; Biotin metabolism; Vitamin C metabolism; Pyrimidine synthesis; Citric acid cycle; CoA synthesis; Transport, mitochondrial; Pentose phosphate pathway; NAD metabolism; Triacylglycerol synthesis; ROS detoxification; D-alanine metabolism; Vitamin A metabolism; Taurine and hypotaurine metabolism; Cholesterol metabolism; Steroid metabolism; Glyoxylate and dicarboxylate metabolism; Thiamine metabolism; Nucleotide interconversion; Androgen and estrogen synthesis and metabolism; Fructose and mannose metabolism; Heme synthesis; Dietary fiber binding; Tetrahydrobiopterin metabolism<br>Valine, leucine, and isoleucine metabolism; O-glycan synthesis; Butanoate metabolism; Sphingolipid metabolism; Vitamin B6 metabolism; Fatty acid synthesis; Vitamin B2 metabolism; Squalene and cholesterol synthesis transport, Nuclear phosphatidylinositol phosphate metabolism; C5-branched dibasic acid metabolism; Glycolysis/gluconeogenesis; Transport, lysosomal; Phenylalanine metabolism; Fatty acid oxidation; N-glycan degradation; Inositol phosphate metabolism; Purine synthesis; N-glycan synthesis; R group synthesis; Keratan sulfate synthesis; Heparan sulfate degradation; Bile acid synthesis; Galactose metabolism; Miscellaneous; exchange/demand reaction |

**Table S3. MRM transitions for free carnitine and acylcarnitines and the corresponding internal standard.**

| Analyte | Analyte | Transition | |
|---|---|---|---|
| | | Q1 (*m/z*) | Q2 (*m/z*) |
| Free carnitine | Carnitine | 218 | 85 |
| | Carnitine-D9 | 227 | 85 |
| Acetylcarnitine | C2-Carnitine | 260 | 85 |
| | C2-Carnitine-D3 | 263 | 85 |
| Propionylcarnitine | C3-Carnitine | 274 | 85 |
| | C3-Carnitine-D3 | 277 | 85 |
| Butyrylcarnitine | C4-Carnitine | 288 | 85 |
| | C4-Carnitine-D3 | 291 | 85 |
| Isovalerylcarnitine | C5-Carnitine | 302 | 85 |
| | C5-Carnitine-D9 | 311 | 85 |
| Glutarylcarnitine | C5DC-Carnitine | 388 | 85 |
| | C5DC-Carnitine-D6 | 394 | 85 |
| Hexanoylcarnitine | C6-Carnitine | 316 | 85 |
| | C6-Carnitine-D3 | 319 | 85 |
| Octanoylcarnitine | C8-Carnitine | 344 | 85 |
| | C8-Carnitine-D3 | 347 | 85 |
| Decanoylcarnitine | C10-Carnitine | 372 | 85 |
| | C10-Carnitine-D3 | 375 | 85 |
| Lauroylcarnitine | C12-Carnitine | 400 | 85 |
| | C12-Carnitine-D3 | 403 | 85 |
| Tetradecanoylcarnitine | C14-Carnitine | 428 | 85 |
| | C14-Carnitine-D3 | 431 | 85 |
| Palmitoylcarnitine | C16-Carnitine | 456 | 85 |
| | C16-Carnitine-D3 | 459 | 85 |
| Stearolycarnitine | C18-Carnitine | 484 | 85 |
| | C18-Carnitine-D3 | 487 | 85 |

**Table S4. MRM transitions for amino acids and the corresponding internal standard.**

| Analyte | Transition | |
|---|---|---|
| | Q1 (*m/z*) | Q2 (*m/z*) |
| Alanine | 146 | 44 |
| Alanine-D4 | 150 | 48 |
| Aspartic acid | 246 | 144 |
| Aspartic acid -D3 | 249 | 147 |
| Glutamic acid | 260 | 158 |
| Glutamic acid -D5 | 265 | 163 |
| Leucine | 188 | 86 |
| Leucine-D3 | 191 | 89 |
| Methionine | 206 | 104 |
| Methionine-D3 | 209 | 107 |
| Phenylalanine | 222 | 120 |
| Phenylalanine-D5 | 227 | 125 |
| Tyrosine | 238 | 136 |
| Tyrosine-D4 | 242 | 140 |
| Valine | 174 | 72 |
| Valine-D8 | 182 | 80 |
| Arginine | 231 | 70 |
| Arginine-D7 | 238 | 77 |
| Citrulline | 232 | 113 |
| Citrulline-D2 | 234 | 115 |
| Glycine | 132 | 76 |
| Glycine-13C2-15N | 135 | 79 |
| Ornithine | 189 | 70 |
| Ornithine-D6 | 195 | 76 |
| Proline | 172 | 116 |
| Proline-D7 | 179 | 123 |

**Figure S1.** OPLSDA model validation including the two groups: MPS VI and Controls. Below the figure, model parameters and CV-ANOVA results are presented.

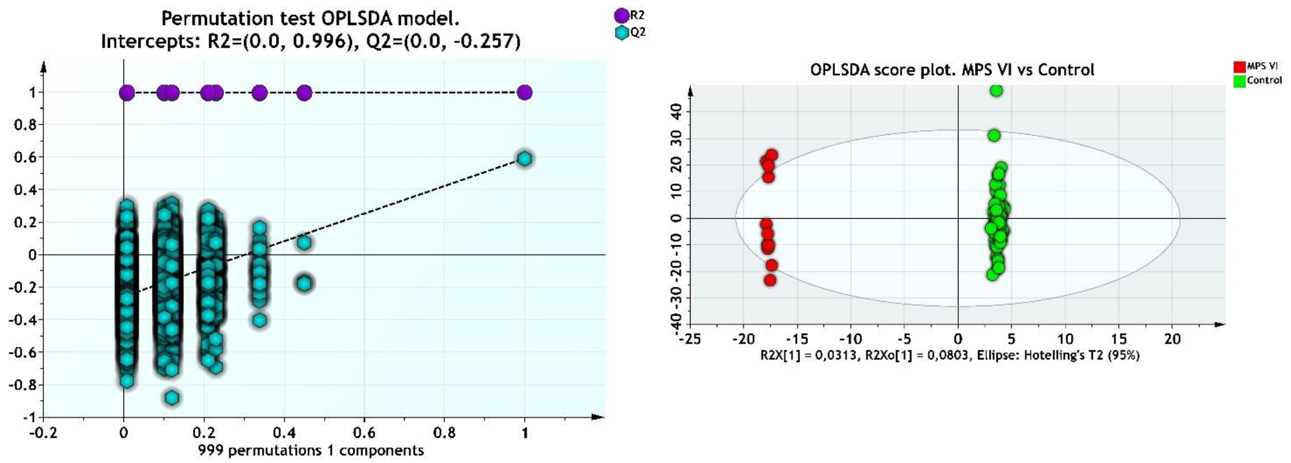


**MPS VI *vs* Controls - OPLSDA model parameters.**

| Component | R2X | R2X(cum) | Eigenvalue | R2 | R2(cum) | Q2 | Limit | Q2(cum) | R2Y | R2Y(cum) |
|---|---|---|---|---|---|---|---|---|---|---|
| Model | | 0.255 | | | 0.999 | | | 0.592 | | 1 |
| Predictive | | 0.0313 | | | 0.999 | | | 0.592 | | 1 |
| P1 | 0.0313 | 0.0313 | 1.97 | 0.999 | 0.999 | 0.592 | 0.01 | 0.592 | 1 | 1 |
| Orthogonal in X(OPLS) | | 0.223 | | | 0 | | | | | |
| O1 | 0.0803 | 0.0803 | 5.06 | 0 | 0 | | | | | |
| | 0.0574 | 0.138 | 3.61 | 0 | 0 | | | | | |
| O2 | 0.0266 | 0.164 | 1.67 | 0 | 0 | | | | | |
| | 0.0328 | 0.197 | 2.07 | 0 | 0 | | | | | |
| | 0.0263 | 0.223 | 1.66 | 0 | 0 | | | | | |

**MPS IV *vs* Controls - OPLSDA model parameters.**

| | SS | DF | MS | F | *p* | SD |
|---|---|---|---|---|---|---|
| Total corr. | 62 | 62 | 1 | | | 1 |
| Regression | 36.7331 | 12 | 3.0611 | 6.05753 | 2.143e-006 | 1.7496 |
| Residual | 25.2669 | 50 | 0.505337 | | | 0.710871 |

**Figure S2. A)** Boxplots of selected discriminant features and dermatan sulfate in the assessed groups: MPS VI and Control samples. B**)** Correlation plot of aminoacids, dermatane sulfate and selected discriminant features. Correlation is displayed in percentage. The size of the circles is correlated to the correlation coefficients.

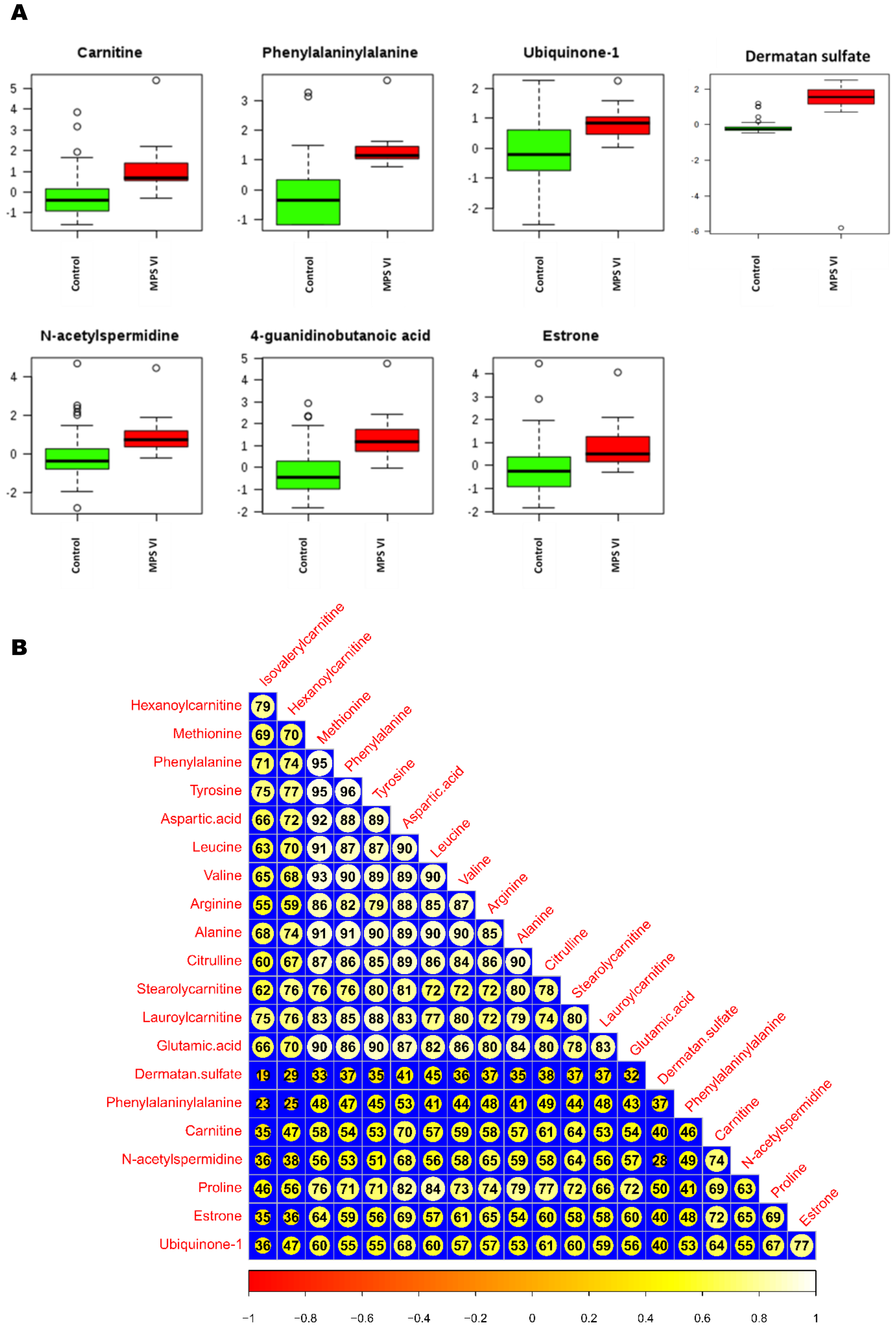

**Figure S3.** Boxplots of amino acid, carnitine (free and total) and acylcarnitine concentrations.

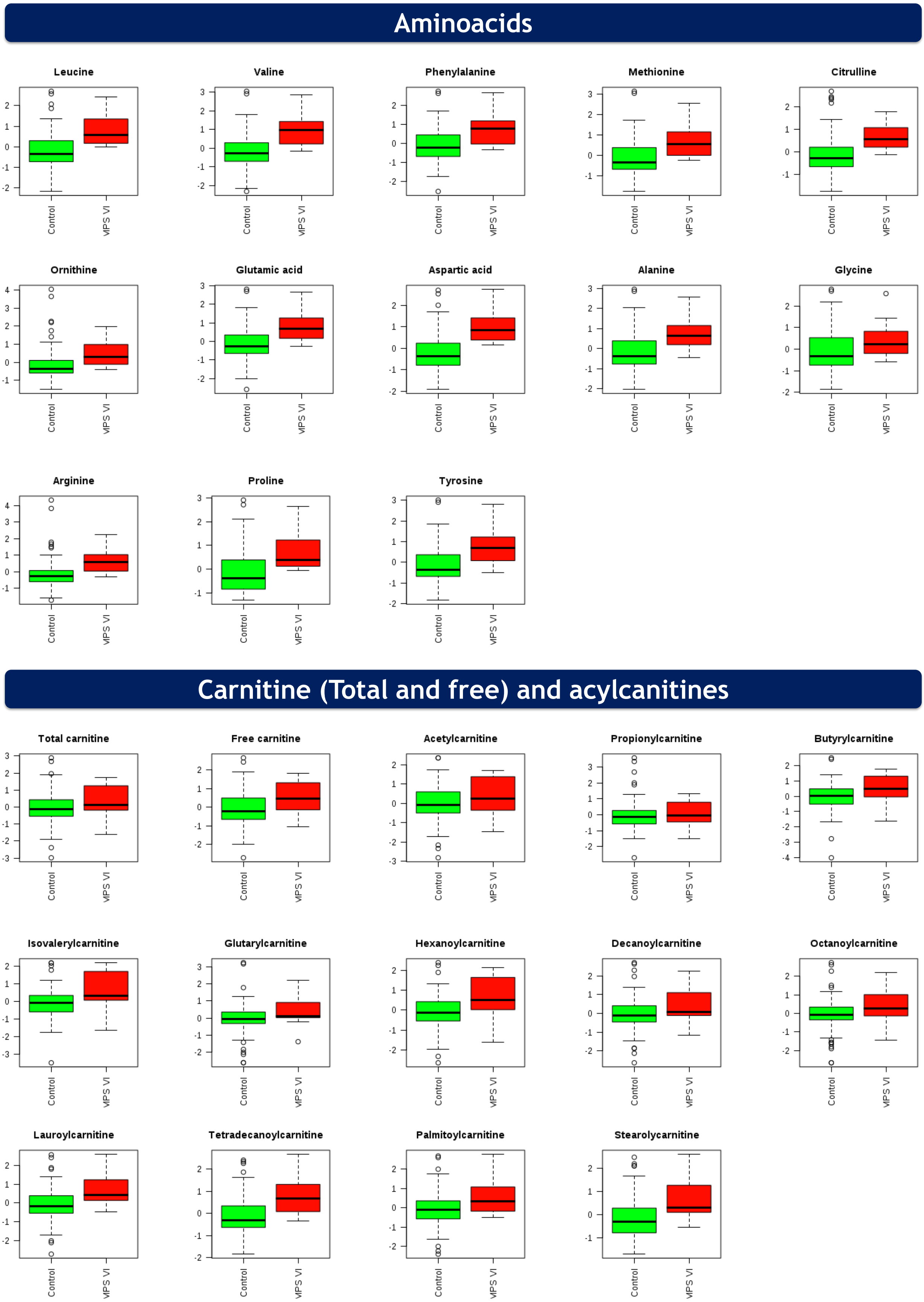

**Figure S4.** The area under the receiver operating characteristic (ROC) curves, comparing diagnostic performance of the most significant quantified amino acids to differentiate MPS VI from Control samples. A) Aspartic acids, Valine and Arginine. B) A comparison of different combinations of seven most significant metabolites using either a PLSDA or Support Vector Machine (SVM) model with three components each is presented. Combining metabolites does not show improvement in AUC.

AUC, Area under the curve. False positive rate = 100 – Specificity. True positive rate = Sensitivity.

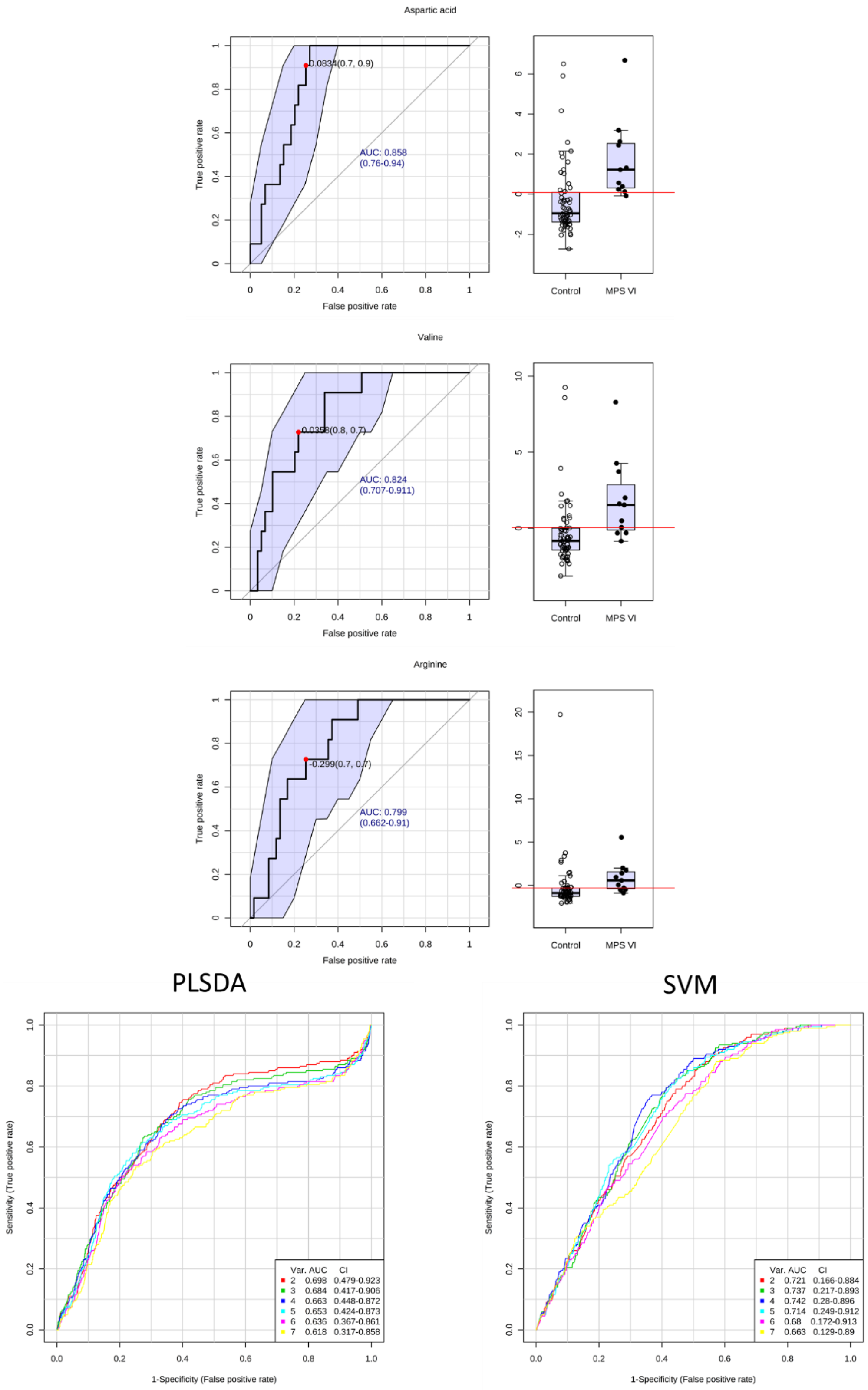

# References

1. Salazar, D.A.; Rodriguez-Lopez, A.; Herreno, A.; Barbosa, H.; Herrera, J.; Ardila, A.; Barreto, G.E.; Gonzalez, J.; Almeciga-Diaz, C.J. Systems biology study of mucopolysaccharidosis using a human metabolic reconstruction network. *Molecular genetics and metabolism* **2016**, *117*, 129-139.
